# Injury-specific effect of Schwann cell-derived exosome treatment for peripheral nerve injury

**DOI:** 10.1371/journal.pone.0340322

**Published:** 2026-02-02

**Authors:** Ericka A. Schaeffer, Emily L. Errante, Samuel Nodal, Lisandra Vazquez Diaz, Adham M. Khalafallah, Aisha Khan, W. Dalton Dietrich, Allan D Levi, Stephen Shelby Burks

**Affiliations:** 1 The Miami Project to Cure Paralysis, University of Miami Miller School of Medicine, Miami, Florida, United States of America; 2 Department of Neurological Surgery, University of Miami Miller School of Medicine, Miami, Florida, United States of America; 3 Interdisciplinary Stem Cell Institute, University of Miami Miller School of Medicine, Miami, Florida, United States of America; Nathan S Kline Institute, UNITED STATES OF AMERICA

## Abstract

Peripheral nerve injury (PNI) is characterized by a loss of cellular and axonal integrity that can lead to limited functional recovery. Because many PNIs are not amenable to repair with traditional techniques, cell therapies have emerged as a treatment option. Exosomes, which can be secreted by Schwann cells (SC), carry cellular signaling molecules that facilitate intercellular communication. Our laboratory and others have success using SC-derived exosomes in preclinical PNI models; however, there is no study that directly compares recovery from different PNIs after exosome treatment. Thus, the currently study investigated if SC-derived exosomes can effectively treat different types of PNI, as measured by axonal regeneration and functional recovery. Adult male Fischer rats were divided into several treatment groups, including nerve transection, reversed autograft, conduit + exosomes, nerve crush, and nerve crush + exosomes. Animals underwent functional assessment through the duration of the experiment and at the conclusion (11 weeks), electrophysiological and histological characteristics were assessed. Results indicate an injury-specific effect of SC-derived exosome treatment. Specifically, exosome treatment improved axon regeneration/myelination, muscle recovery, and gait characteristics for severe, large-gap injuries. These SC-exosome based effects were not observed in crush injuries. Taken together, the results of the current study indicate that there may be differences in recovery based on injury type after PNI and treatment with SC-derived exosomes, potentially due to differences in exosome retention/distribution at the injury site. Future studies should explore how exosomes are distributed following administration across various PNI models, as their therapeutic effects may be more pronounced in upstream regions.

## Introduction

Peripheral nerve injury (PNI) is a common condition resulting in a heterogenous set of symptoms, depending on injury severity. More severe injuries have damage to the axon and surrounding structures, creating gaps in the peripheral nerve which can be larger than the nerve’s ability to regenerate (4 cm humans, 10–15 mm in rats) [1–3]. Conversely, crush PNI can damage the axon without completely transecting surrounding structures. As there are distinctions in symptoms and natural recovery, determining treatment course also varies based on injury severity. For example, large gap PNI typically require surgical intervention (e.g., direct repair, nerve transfer/graft, guidance channel). Treatment of less severe injuries mostly include palliative care and symptom management. With current treatment options, functional recovery is gradual and, in some cases, still sub-optimal [4].

Cellular therapies may provide a universal solution for improving regeneration and functional outcomes following PNI. Decades of research have harnessed the therapeutic value of SC following PNI in rodents [5–7], non-human primates [8], and humans [9–12]. Although this cellular treatment is successful, SC implantation requires cell harvesting or cell-donor matching; thus, immunogenicity and potential rejection must be taken into consideration. Recently, research has focused on extracting the therapeutic properties of cells critical for regeneration following PNI, termed exosomes. Exosomes are small extracellular vesicles that are released from a variety of cells containing gene-modifying properties which can interact with neighboring cells, regulating cell-to-cell communication [13–17]. SC exosome-based therapies in PNI successfully improve regeneration preclinically [18–22]. Our lab has previously found therapeutic efficacy in large-gap (15 mm) PNI with nerve guidance channels, or conduits, loaded with human SC-derived exosomes [23,24]. Specifically, axonal myelination quantities, in addition to sensory outcomes, were similar to the gold standard treatment, autograft [24]. Although our lab has not assessed SC-derived exosomes in less severe injuries (i.e., crushes), others have found accelerated regeneration 4-days post injury/treatment [19]. However, this study did not evaluate functional outcomes or long-term changes.

Despite encouraging findings, numerous areas still require further investigation to determine therapeutic translatability across PNI. Source of exosome is critical to consider. Much of the current literature evaluating SC-derived exosomes utilizes stem cells altered to have the SC-like phenotype [25]. SC-derived exosomes are observed to be more effective in treating PNI compared to those derived from mesenchymal stem cells [17,21,26,27]. Additionally, it remains unclear whether exosomes derived from SCs of the same species (human vs. rat) produce differing effects on histological and functional outcomes, as our lab has only studied human SC-derived exosomes previously [24]. Typically, crush PNI studies endpoints are short term. As such, the long-term effect of SC-derived exosome therapy across different levels of PNI severity has yet to be examined. Therefore, the current study investigated the longitudinal effects (over 11 weeks) of rat SC-derived exosome treatment following either severe (15 mm gap) or mild (crush) PNI.

## Methods

### SC-derived exosomes

Rat SC-derived exosomes were acquired from available cultures provided by the Miami Project to Cure Paralysis cellular laboratory. Briefly, exosomes were isolated from primary rat SC cultures that were sourced from adult, female Fischer rat sciatic nerves (Envigo). Rat SC cultures were grown using techniques as previously described [24,28–30]. Following growth, cultures were washed and then underwent ultracentrifugation (centrifuge at 3g for 10 min, filtered with a 0.22μM cellulose acetate filter (Corning), centrifuge at 100,000g for 130 min) until the supernatant containing the exosomes was aspirated [28]. Exosomes were confirmed through vesicle size distribution and composition as previously reported [28]. Exosome treatment consisted of a single 350 μL injection surrounding the exposed sciatic nerve or loaded conduit, depending on group condition, at a concentration was 6.66x10^11^ particles/mL, resulting in approximately 2.33x10^11^ total exosomes injected. This high concentration/dose was selected based on observed therapeutic efficacy in large-gap PNI [24].

### Animals and PNI models

**Fig 1:**
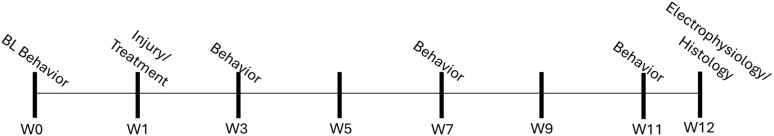
Timeline of Experiment: A timeline of behavioral tests, surgical/treatment intervention, and electrophysiological/histological endpoints are displayed.

Sixty adult male Fischer rats (*Rattus Fisher*; Envigo; n = 12) were used in this study. Animals were pair housed with corncob bedding and were kept on a 12-h light/dark cycle. Two rats were excluded from the study: one did not recover from surgery, and the other was euthanized due to excessive self-mutilation resulting in a total of 58 rats. All procedures were approved by the Institutional Animal Care and Use Committee (IACUC) at the University of Miami. All animals had *ad libitum* access to food and water. They were housed in the veterinary facilities of our institution and were cared for according to the NIH *Guide for the Care and Use of Laboratory Animals*. Animals were randomly assigned into one of five experimental groups: 13 mm nerve transection (n = 11; negative control), reverse autograft (n = 11; positive control), conduit + exosomes (n = 12), nerve crush control (n = 12; negative control), and nerve crush + exosomes (n = 12). An empty conduit group was not included, as our prior work has compared conduits loaded with SC-derived exosomes with the same defect length, rendering the group redundant [6,24]. Therefore, a true negative control (transection, no repair) was included in the current study, as this has yet to be evaluated. The survival time for all rats in the study was 12 weeks after surgery (Fig 1).

Our surgical technique has been previously described [6,8,11,31,32]. Briefly, all animals were anesthetized and then had their right leg shaved prior to the start of surgery. A posterior incision from the right iliac crest to the knee joint was made under aseptic conditions. The bicep femoris was reflected and the sciatic nerve beneath was dissected from the surrounding tissue. For the transection, autograft, and conduit groups a 13 mm segment of nerve was removed via sharp transection distal to the notch and proximal to the bifurcation. For the reverse autograft, the transected nerve was rotated 180-degrees and sutured to the distal/proximal stumps using an operating microscope with 10−0 nylon sutures (Ethicon). The conduit implants consisted of NeuraGen 3D nerve guide (Integra Lifesciences Corp.) [6,24,33,34]. These nerve guides have an internal diameter of 1.5 mm and a length of 15 mm and were loaded with the SC-derived exosomes as described [23,24]. Crush injuries were modeled after previous research [19,35–40]. Briefly, the midpoint of the exposed sciatic nerve was estimated, and Dumont forceps were applied to the nerve perpendicularly with pressure applied for 10s, then removed for 10s. This procedure was repeated for a total of three times (30s total crush time). For the crush + exosome group, SC-derived exosomes were injected (350 μL) around the nerve. For corresponding injury/treatment, the wound was closed in layers with 4−0 polyglactin sutures (Webcryl) and the skin was stapled. Staples were removed postoperative day 7. Immediately after surgery, all animals were treated with buprenorphine for pain. Apple bitter spray was used prophylactically for self-mutilation. Nylabones were added to all cages.

### Sensory function

#### Mechanical nociception.

Calibrated von Frey filaments were applied sequentially with increasing force to the plantar skin of both the hind paws, using the Dixon up-down method [41,42]. This procedure was evaluated prior to injury as baseline (BL) and reoccurred at several timepoints (3, 7, and 11 weeks) following surgery. As a comparative control, the percent difference between the injured and uninjured limbs was calculated from the Dixon scores.

#### Cold nociception.

To assess cold stimulus sensitivity, the acetone test was conducted [43]. This procedure was evaluated prior to injury as BL and recurred at several timepoints (3, 7, and 11 weeks) following surgery for both limbs. As a comparative control, the percent difference between the injured and uninjured limbs was calculated from the response frequency scores.

#### Heat nociception.

Rats underwent the Hargreaves test to evaluate reaction time to a heat source [44]. This procedure was evaluated prior to injury as BL and recurred at several timepoints (3, 7, and 11 weeks) following surgery for both limbs. As a comparative control, the percent difference between the injured and uninjured limbs was calculated from the reaction times.

### Motor function

#### Sciatic functional index (SFI).

Toe spread and print length were assessed during walking at 3, 7, and 11 weeks post-injury to calculate SFI [45].

#### Gait.

To assess changes in pain-related limitations/compensations in mobility, gait was assessed based on previous research [46]. The apparatus consisted of a clear, Plexiglass runway and rats walked the runway for a total of three trials. Limb movements were recorded from below at 30 frames/second (Canon XA40) for offline analysis. The three trials/videos of each rat were cropped to exclude any instances of grooming, rearing, or prolonged stopping. Then, the hind limbs were manually labeled frame-by-frame for each trial using Tracker (https://physlets.org/tracker/). The tracked points were divided into phases of gait: swing (limb is in motion) and stance (limb is not in motion). As the limb is not in motion during the stance phase, the swing phase was analyzed to derive several kinematic and topographic measures of gait based on previous work [47–49], including peak speed, distance traveled, limb path circuity, and peak error. During normal gait, limbs move in direct trajectories from location a to location b. The ratio between the actual path taken by the limb and the most direct route, Euclidean distance, reflects variability in limb movement called limb path circuity [46,50,51]. Typically, when animals are accurately estimating distance, their fastest speeds should occur at the midpoint [50,52]. Peak error measures the variability in the location at which the peak speed occurs when the limb is moving. Values closer to 0 indicate peak speeds occurring at the midpoint, whereas values closer to 0.5 indicate peak speeds occurring at the start or end of the limb movement (initiation/termination). Gait was assessed and analyzed at several timepoints (3, 7, and 11 weeks) following surgery for both limbs. As a comparative control, the percent difference between the injured and uninjured limbs was calculated from the response gait characteristics.

#### Electrophysiology.

To assess neuromuscular recovery following PNI, gastrocnemius electrophysiological characteristics (action potential amplitude and onset) of both the injured and uninjured limb were evaluated using the tendon-belly method [53,54] at the endpoint of the experiment (11 weeks). Rats were deeply anesthetized, and monopolar needle electrodes were inserted into the belly of the gastrocnemius muscle and Achilles tendon. Ground electrodes were inserted percutaneously into the neck. The sciatic nerve was stimulated with a bipolar electrode placed over the sciatic notch. Data was recorded with Digitimer acquisition hardware and processed offline using Cambridge Electronic Design (CED) Signal v8.1. As a comparative control, the percent difference between the injured and uninjured limbs was calculated from the electrophysiological characteristics.

#### Muscle weight.

At 12-weeks post-injury, rats were sacrificed and perfused. Following perfusion, an incision was made from the sciatic notch down to the paw on both limbs. The gastrocnemius was then harvested via sectioning of the proximal and distal tendinous attachments. This was repeated on both legs. The gastrocnemius from each leg was then weighed dry on a precision balance to assess percent muscle recovery.

### Histology

Following active perfusion, the sciatic nerves from the injured limb were harvested and sliced. One-micrometer plastic cross sections (n = 20) were stained with 1% toluidine blue (TB), 1% methylene blue, and 1% sodium borate solution. Sections were analyzed using an Olympus BX51 microscope at 100x magnification and Stereoinvestigator software (version 2019.1.2, MBF Bioscience) with an Optical Fractionator. The cross-sectional area of the epineurium was estimated by point counting with a 20x dry objective. A sampling grid of 134 μm x 134 μm with a counting frame of 30 μm x 30 μm was chosen. The total number of myelinated axons was extrapolated from the product of the area density and cross-sectional area. The randomly selected subset of counted axons was also used to determine axon diameter and myelin thickness using the Stereoinvestigator measure line tool (MBF Bioscience). Axon diameter in relation to myelin thickness was quantified as a G-ratio. Unbiased stereological analysis was completed by three blinded investigators and averaged per animal and per group.

### Statistical analysis

Separate repeated-measure ANOVAs were performed to assess the main effect of treatment condition, within-subjects variable (timepoint), and their corresponding interactions. All data presented are represented by means and standard deviations. Orthogonal contrasts were used for post-hoc and exploratory analyses. All analyses were processed through JASP 18.1 (University of Amsterdam) statistical software with an alpha cut-off of 0.05.

## Results

### Sensory function

**Fig 2:**
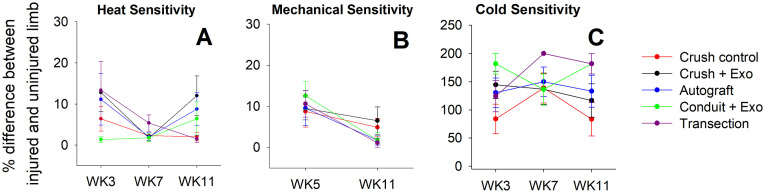
Sensory Testing Reveals no Treatment-Related Effects: Heat (A), mechanical (B), and cold (C), sensitivity difference scores, between the injured and uninjured limb, are plotted for each injury and treatment condition across the duration of the study (11 weeks). Heat sensitivity significantly varied across time with a quadratic trend showing mid-study normalization, while cold and mechanical sensitivity showed no group effects, except for a significant reduction in mechanical sensitivity differences across time in the crush + exosome group. [n = 12 crush control; n = 12 crush + exosome; n = 11 autograft; n = 11 conduit + exosome; n = 12 transection].

A battery of behavioral tests was conducted to detect changes to sensory nociception. The uninjured limb was evaluated in the following behavioral tasks for comparative purposes, as represented by the percent difference between injured and uninjured limbs. Differences in heat sensitivity, measured by Hargreaves test, was significantly different across testing timepoints following a Greenhouse-Geisser correction for sphericity (Fig 2A) [F (1.78, 94.52), = 5.55, p = 0.007, η_p_^2^ = 0.095]. Trend analysis contrasts revealed a significant quadratic trend, with rats displaying more similar heat reaction times between limbs at the midpoint of the study (WK7) [p = 0.002]. Cold (acetone response) and mechanical (Von Frey filaments) sensitivity tests failed to reveal differences between groups and across time (Fig 2B,C). Although no main effects were observed, exploratory orthogonal contrasts were conducted to compare the first timepoint (WK3) to the last timepoint (WK11). The crush + exosome group had a significant reduction in the difference between limbs for mechanical sensitivity across the duration of the study (WK7: M = 25.93% difference, SD = 41.38%; WK11: M = 0% difference, SD = 0%) [t(53) = 3.23, p = 0.002]. The remaining groups did not show a similar trend of improvement between the injured limb across time.

### Motor function

**Fig 3:**
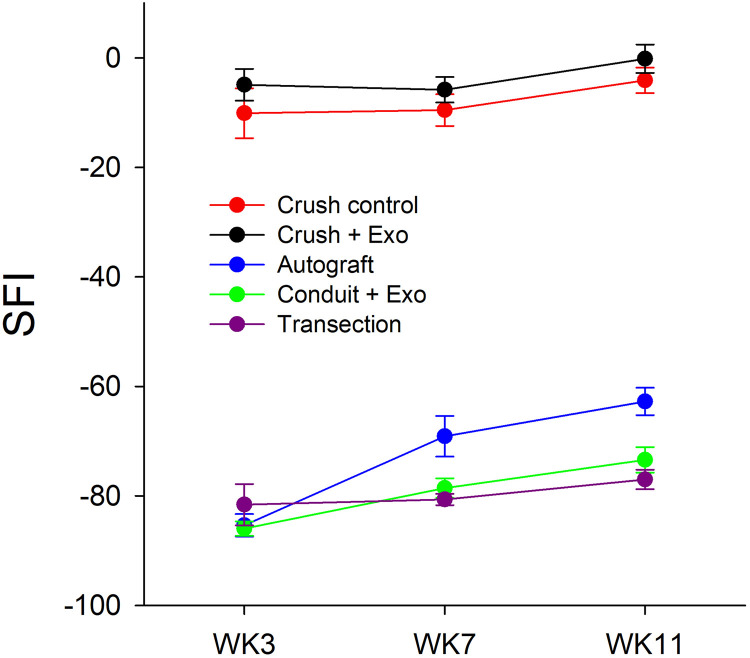
SFI Improves only in Autograft & Conduit + Exosome Groups: Sciatic functional index (SFI) is plotted across week 3, 7, and 11 for each injury and treatment condition. SFI analyses revealed significant time, group, and interaction effects, with only the autograft and conduit + exosome groups showing functional improvement from WK3 to WK11. [n = 12 crush control; n = 12 crush + exosome; n = 11 autograft; n = 11 conduit + exosome; n = 12 transection].

Several measures were evaluated to assess functional recovery following sciatic nerve injury. SFI was calculated at WK3, WK7, and WK11 post-injury and analyses revealed a significant effect of testing timepoint [F(2, 106) = 19.38, p < 0.001, η_p_^2^ = 0.27], group [F(4, 53) = 516.29, p < 0.001, η_p_^2^ = 0.98], and corresponding interaction [F(8, 106) = 2.89, p = 0.006, η_p_^2^ = 0.18] (Fig 3). Between WK3 and WK11, only the autograft (∆M = 23.21) and conduit + exosome (∆M = 12.54) group had significant improvements in SFI [p < 0.001; p = 0.002, respectively]. The transection and crush groups did not display improvements in SFI across time.

**Fig 4:**
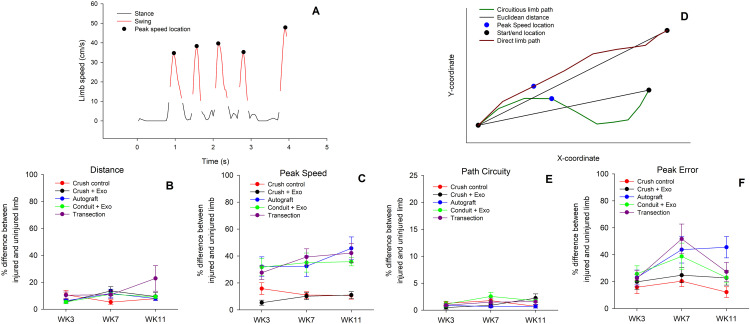
Direct Gait Movements Observed Across Groups: The kinematics of gait are plotted for a representative rat (A). Gait is typically broken up into two sequential phases based on speed including swing (A, red line) and stance (A, black line). The difference between the injured and uninjured limb’s distance **(B)** and peak speed **(C)** during the swing phase is plotted across 3, 7, and 11 weeks for each injury/treatment condition. The transection group showed greater overall limb-distance differences than the autograft and conduit groups and was the only group to exhibit worsening asymmetry over time. Peak speed differences between limbs significantly varied by group, with larger asymmetries in gap-injury groups than crush groups. The topography of limb movement during gait is plotted for a representative rat **(D)**. The directness of limb movement, or path circuity, during the swing phase can be represented as a ratio between the actual path taken and the Euclidean distance. An example of a direct (D, dark red line) and circuitous (D, green line) are plotted along with the corresponding Euclidean distance (D, black line). Typically, when animals are accurately estimating distance, their fastest speeds (D, blue dot) should occur at the midpoint, measured as peak error. The difference between the injured and uninjured limb’s path circuity **(E)** and peak error **(F)** during the swing phase is plotted across 3, 7, and 11 weeks for each injury/treatment condition. Limb path circuity showed no significant differences across groups or time. Peak error differed by group and time, driven by increasing asymmetry in the autograft group, which exceeded the conduit + exosome group at the final timepoint. [n = 12 crush control; n = 12 crush + exosome; n = 11 autograft; n = 11 conduit + exosome; n = 12 transection].

For a more nuanced characterization of limb function, kinematic and topographic gait characteristics were analyzed. During walking, gait is typically divided into two phases based on speed: swing (i.e., when the limb is in movement) and stance (i.e., when the limb is stationary) (Fig 4A). The peak speed of limb moment typically occurs at the midpoint of the swing phase (Fig 4A). Since the limb does not display meaningful kinematic changes during the stance phase, only the swing phase was analyzed for the following measures. The difference between limbs’ distance traveled during the swing phase did not reveal significant main effects (Fig 4B). Exploratory orthogonal contrasts were conducted and the transection group (M = 14.67%, SD = 19.76) was found to have overall greater differences between injured/uninured limbs, compared to the autograft (M = 8.59%, SD = 7.54) and conduit group (M = 8.55%, SD = 4.60) [p = 0.023; p = 0.025 respectively]. Additionally, the transection group was the only one to increase the difference in limb distance across time between WK3 (M = 10.39%, SD = 7.91) and WK11 (M = 22.87%, SD = 31.35) [p = 0.010]. Other general characteristics of gait include peak speed, which revealed a significant effect of group (Fig 4C) [F(4, 53) = 21.82, p < 0.001, η_p_^2^ = 0.62]. In general, there were greater changes in peak speed between the limbs for the gap groups (autograft/transection/conduit), compared to the crush groups [all p < 0.05]. There were no noted differences within the gap and crush groups. Additionally, contrasts were conducted to evaluate group changes over time. Between WK3 and WK11, only the transection (∆M = 14.53%) and autograft (∆M = 13.59%) groups displayed greater differences in peak speed between limbs.

Typically, during the swing phase of gait, limbs make direct, ballistic trajectories (Fig 4D). The circuitousness of a limb’s path during the swing phase can be evaluated as a ratio between the actual path taken and the Euclidean distance (i.e., shortest distance between start/end location). Representative direct (red line) and circuitous (green line) limb swing paths are plotted for a rat, along with the Euclidean distance (black lines) (Fig 4D). The difference between limb path circuity did not reveal any significant effects (Fig 4E). In general, the rats displayed direct gait movements. The distance of the limb path is found to be related to the peak speed and the location where that peak speed occurs. Typically, when animals are accurately estimating distance, their fastest speeds should occur at the midpoint, which is characterized by the peak error measure (Fig 4D). The difference in peak error values between limbs was found to be significantly different between groups [F(4, 53) = 4.60, p = 0.003, η_p_^2^ = 0.26] and across timepoints [F(1.73, 91.90) = 6.30, p = 0.004, η_p_^2^ = 0.11] with a Greenhouse-Geisser sphericity correction (Fig 4F). Differences in peak error values appeared to be similar across time for all groups, with one exception. The autograft group displayed greater differences in peak error values from WK3 (M = 23.10%, SD = 19.13) to WK11 (M = 45.55%, SD = 27.41) [p = 0.003]. At the final timepoint (WK11), the autograft group had significantly larger deviations in peak error between limbs compared to the conduit + exosome group (M = 23.05%, SD = 13.13) [p = 0.009].

**Fig 5:**
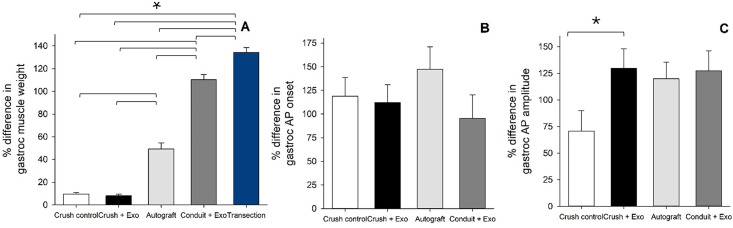
Muscle Weight Asymmetry Varies by Treatment, but Action Potential Onset and Amplitude Do Not: The gastrocnemius muscle weight of the injured and uninjured limb was assessed at the conclusion of the study (A). Several significant differences between treatment conditions were noted **(A)**. Electrophysiology was also conducted at the conclusion of the study and compared between injured and uninjured limbs. Action potential (AP) onset (B) and amplitude (C) are plotted for each injury and treatment condition. [Note: * indicates p < 0.05; n = 12 crush control; n = 12 crush + exosome; n = 11 autograft; n = 11 conduit + exosome; n = 12 transection].

The gastrocnemius muscle weight and electrophysiological characteristics (e.g., action potential amplitude/action potential onset) were compared between injured and uninjured limbs at the conclusion of the study (WK11). Regarding gastrocnemius muscle weight, there was an observed effect of group (Fig 5A) [F(4, 53) = 239.84, p < 0.001, η_p_^2^ = 0.95]. Post-hoc orthogonal contrasts revealed several differences with the transection group displaying the greatest difference in weight between limbs, compared to all other groups [all p < 0.001]. Additionally, the conduit + exosome group had significantly larger differences in muscle weight compared to the autograft and crush groups [all p < 0.001]. The autograft group displayed larger deviations in muscle weights between limbs, compared to the two crush groups [all p < 0.001]. The crush control and crush + exosome group did not significantly differ [p = 0.75]. The transection group was not included in the electrophysiological results, as no signal was observed due to lack of sciatic nerve. The difference between limbs in gastrocnemius muscle action potential onset (Fig 5B) and amplitude (Fig 5C) did not reveal a significant effect of group.

### Histology

**Fig 6:**
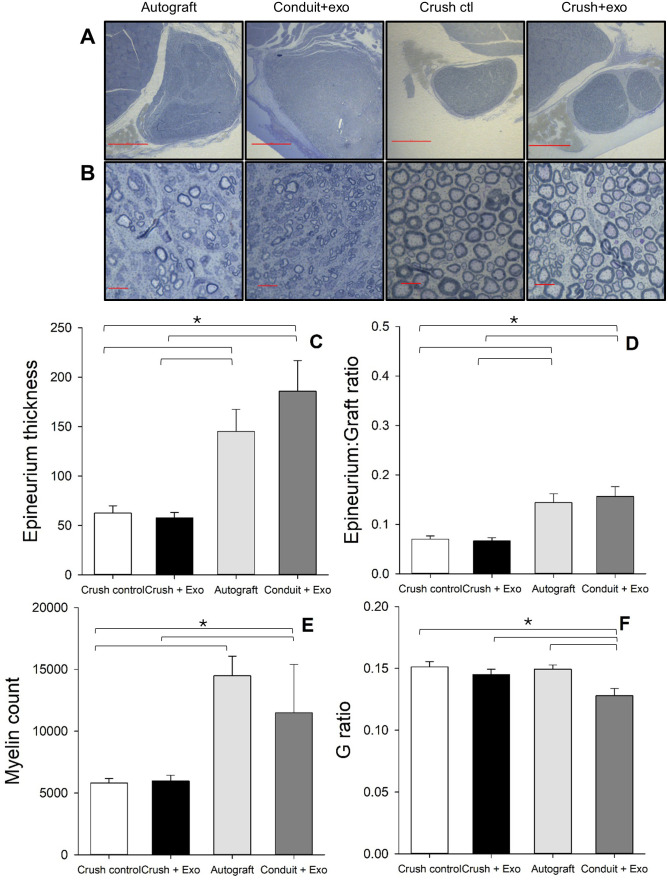
Improved Myelination Profiles in Autograft and Conduit + Exosome Groups, Relative to Crush Groups: Sections of the nerve from the injured limb were stained with tubulin blue to assess remyelination at the conclusion of the study. Representative images of the nerve at 4x **(A)** and myelin rings at 100x **(B)** are displayed for each group. The epineurium thickness **(C)**, epineurium thickness to graft diameter ratio **(D)**, myelin count **(E)**, and myelin thickness to axon diameter (G ratio, **F**) are plotted for each injury and treatment condition. Several differences were noted in these measures between groups. [Note: magnification scale bars represent 500μm (A) and 10μm (B;, * indicates p < 0.05; n = 12 crush control; n = 12 crush + exosome; n = 11 autograft; n = 11 conduit + exosome].

Following the completion of the study, the sciatic nerve was dissected and stained for myelin (Fig 6A & B). As the transection animals did not have a nerve present, they were excluded from histological analyses. First, the width of the epineurium was evaluated and revealed a significant group effect (Fig 6C) [F(3, 42) = 11.32, p < 0.001, η_p_^2^ = 0.45]. The autograft (M = 145.04 μm, SD = 77.57) and conduit + exosome (M = 185.79 μm, SD = 97.86) group displayed thicker epineurium’s than the crush groups. No differences were noted between the autograft and conduit group. In addition, the ratio between the epineurium thickness and graft diameter was evaluated. There was a significant group effect [F(3, 42) = 11.95, p < 0.001, η_p_^2^ = 0.46] of epineurium to graft ratio (Fig 6D). Both the autograft (M = 0.14, SD = 0.063) and conduit + exosome (M = 0.16, SD = 0.063) displayed greater epineurium thickness in relation to total graft diameter compared to the crush groups. No differences were noted between the autograft and conduit group.

The number of myelinated axons were found to significantly vary between groups (Fig 6E) [F(3, 42) = 5.16, p = 0.004, η_p_^2^ = 0.30]. The autograft group (M = 14477.023, SD = 5505.87) had more myelinated axons than the crush control (M = 5800.37, SD = 1589.41) [p = 0.002] and crush + exosome (M = 5987.71, SD = 1531.71) [p = 0.003] groups. Additionally, the conduit + exosome group (M = 11481.87, SD = 3917.28) displayed more myelinated axons compared to crush controls [p = 0.047]. The conduit and autograft groups did not significantly differ in myelin counts. G-ratio analysis revealed a significant effect of group (Fig 6F) [F(3, 42) = 5.18, p = 0.004, η_p_^2^ = 0.27]. The conduit + exosome group was found to have the smallest G ratio [all p < 0.05], indicating they had thicker myelin in relation to axon diameter.

## Discussion

This study investigated the long-term therapeutic efficacy of rat SC-derived exosomes in two distinct PNI models, severe (15 mm gap) and mild (crush), with a focus on axonal regeneration and functional recovery. Our results demonstrate a clear injury-specific effect of exosome treatment, with significant benefits observed in severe gap injuries but limited impact in crush injuries. These findings highlight the nuanced role of exosome-based therapy in PNI and underscore the need for injury-contextualized approaches in future clinical translation.

Consistent with prior work from our group [24], we found that SC-derived exosomes promoted robust regeneration in the conduit model, evidenced by increased myelinated axon counts and improved myelin thickness. Notably, the conduit + exosome group demonstrated functional outcomes approaching those of the autograft group, the current clinical gold standard. These findings are exciting as there is variability/limitations in donor nerve sources [55], typically resulting in compromised function in additional areas for clinical autografts. Further, the results from the current study evaluating rat-derived SC exosomes converge with our previous studies [24] that assessed human-derived SC exosomes, which is essential for replication purposes. In contrast, exosome treatment did not enhance recovery in the crush injury model. Neither functional nor histological outcomes were significantly improved in the crush + exosome group compared to the crush control, contrasting previous work [56,57]. For exploratory purposes, we qualitatively compared our historic empty conduit behavioral and histological data to the conduit + exosome data in the current study, respectively. In terms of myelination, the conduits loaded with rat SC-derived exosomes displayed smaller g ratios (0.128 vs. 0.197) [24], thicker axon diameter (4.28μm vs. 3.21μm) [24], and fewer quantities of myelinated axons (11481.87 vs. 19277.76) at the distal end. However, it is important to note the historic histology data was captured at 16-weeks post-injury/repair. For sensory testing at 12-weeks post-injury, the conduit + exosome group displayed slower reaction times to heat (19.75s vs. 6.35s) [24] but displayed similar scores on mechanical sensitivity (Dixon up-down method: 15 vs.15) [24]. In sum, the findings of the current study suggest that the regenerative benefit of exosomes may be context-dependent, leading us to consider potential mechanisms underlying these differences.

Multiple factors may explain the discrepancy in exosome efficacy between injury models, including source, biodistribution, and injury severity. First, exosome source and administration may be directly tied to efficacy. The previously mentioned crush injury studies utilized exosomes derived from stem cells [37,56–58] or stem cells with a SC-like phenotype [59]. These studies failed to evaluate long-term outcomes; therefore, based on the current studies’ results, the therapeutic efficacy in mild PNI remains unclear. Additionally, previous studies have shown that exosomes derived from repair-phenotype SCs can enhance repair specific cargo [17,60]. In the present study, we did not manipulate SCs toward a repair phenotype, as a homogeneous and reproducible population of cultured SCs was necessary to directly compare injury-specific responses and maintain consistency with previously published human SC exosome work from our laboratory [24]. It remains possible that some of the pronounced differences observed between PNI severities in this study could be attenuated using exosomes derived from repair-phenotype SCs. A longitudinal dosage study and direct comparison of SC-derived, stem cell-derived exosomes, and corresponding repair phenotypes is essential. Second, there may be variability in the biodistribution of exosomes depending on injury. Exosomes travel to upstream areas, including the spinal cord and other organ systems, when administered intravenously [61,62], intrathecally [63], and directly to the injury site [64]. For crush injuries, exosomes may be influencing inflammation at the level of the spinal cord, which isn’t resulting in drastic changes to the injury site/behavior. Future research should evaluate the biodistribution of exosomes in various injuries, as there could be an injury-specific exosome distribution. Lastly, the natural regenerative capacity in mild injuries may be sufficient to mask any added benefit of single dose exosome treatment.

Despite these promising findings, several limitations should be acknowledged. This study did not evaluate the biodistribution or retention of exosomes at the injury site, which may differ significantly between gap and crush injuries. Future studies incorporating labeled exosomes or *in vivo* imaging techniques could provide insight into differential accumulation and uptake. Additionally, while rat SC-derived exosomes were used here to address species matching, future studies should compare exosomes derived from different sources (e.g., human vs. rat) within the same injury model to assess species-specific efficacy. Unfortunately, the current study did not look at the histology immediately following crush injury to characterize specific severity. Crush injury models can be variable, and these models have yet to be compared and classified by injury severity. Future research should comprehensively evaluate crush injury models and their corresponding histological injuries. Additionally, exosome cargo was not characterized in the current study. Prior work from The Miami Project to Cure Paralysis cellular laboratory has extensively profiled the molecular contents of human SC–derived exosomes, including proteomic, miRNA, and lipidomic analyses [28]. Notably, they identified the top 30 expressed miRNA sequences (e.g., miR-21-5p) are involved in key signaling pathways for neural regeneration and neuroprotection. Given that exosomal cargo can vary by species and biological source, differences between human and rat SC–derived exosomes are likely. Other labs have evaluated the cargo of rat SC-derived exosomes using similar isolation techniques to the current study and found high expression of proteins associated with axon regeneration (e.g., carboxypeptidase E, fatty acid-bind protein 5, fibronectin, etc.) and inflammatory inhibition (e.g., Galectin-1, αB-Crystallin, etc.) [65]. Although rats and humans share substantial genomic similarity [66,67], species-specific differences in exosomal cargo are still expected. Accordingly, a separate study is planned to characterize the proteomic, miRNA, and lipidomic contents of exosomes derived from the rat SC cultures used in the present work, enabling direct comparison across multiple rat biologic samples and human-derived exosomes [28].

Exosome-based therapies for peripheral nerve injury show strong promise in preclinical models, but several practical challenges hinder translation to the clinic [68]. Major hurdles include the difficulty of scalable manufacturing, as producing clinically relevant quantities of exosomes with consistent quality remains technically demanding, and current isolation methods often yield preparations with variable purity and heterogeneity [69]. Ensuring long-term storage stability is another limitation, since exosomes may lose bioactivity during freezing–thawing cycles or prolonged storage [70], necessitating improved preservation strategies. Efficient and targeted delivery to injured peripheral nerves also remains a challenge due to rapid systemic clearance and limited tissue penetration. Additional considerations such as standardized quality control, optimal dosing and route of administration, and immunogenicity and regulatory requirements further complicate clinical advancement [71]. Ongoing efforts, such as developing scalable bioreactor-based production systems [72], implementing standardized purification protocols, optimizing lyophilized or otherwise stabilized formulations, and engineering vesicles for enhanced targeting, may help overcome these barriers and facilitate successful clinical translation. With continued innovation and collaboration across disciplines, exosome-based approaches have the potential to evolve from experimental tools into transformative therapies for patients with PNI.

In summary, SC-derived exosome treatment resulted in marked histological and functional improvements in severe PNI, but not in mild injury. These findings suggest that exosome therapy may have selective efficacy based on injury severity, possibly due to differences in natural recovery capacity or exosome biodistribution. Importantly, this study utilized rat SC-derived exosomes, addressing a major gap in the literature, which often relies on SC-like cells derived from mesenchymal stem cells. Prior studies have shown superior regenerative capacity with SC-derived exosomes in PNI, yet few have directly compared their efficacy across injury types or assessed long-term outcomes. Our results provide evidence that exosome efficacy may not be uniform across injury contexts, which is critical for understanding the limitations of SC-derived exosome based therapy. This work advances our understanding of exosome-based peripheral nerve repair strategies and lays the foundation for future studies aimed at optimizing delivery/dosing across varied injury models.
